# Influence of the Period of Abstinence on Semen Quality in a Patient with Systemic Lupus Erythematosus: A Case Report and Review of the Literature

**DOI:** 10.1155/2020/9296858

**Published:** 2020-03-07

**Authors:** Tarah H. B. Waters, Carol Pui Shan Chan, Tin Chiu Li, David Yiu Leung Chan

**Affiliations:** Assisted Reproductive Technology Unit, Department of Obstetrics and Gynecology, Faculty of Medicine, The Chinese University of Hong Kong, Hong Kong, China

## Abstract

Systemic lupus erythematosus (SLE) is a chronic inflammatory disease that can affect fertility. There is currently little information regarding the semen profile of males with SLE. Moreover, there is no consensus on an appropriate period of sexual abstinence for semen analysis and on the use of DNA fragmentation assay, together with multiple semen analyses to document the semen profile in this clinical population. In this case report, multiple semen analyses, with DNA fragmentation assays, were performed for a male with SLE undergoing fertility treatment at our andrology unit. A 4-day period of abstinence improved the semen concentration, total sperm count, total progressive motile sperm, and sperm morphology, with minimal DNA fragmentation. In conclusion, multiple semen analyses obtained after different periods of sexual abstinence, together with DNA fragmentation assays, may be useful to develop a semen profile for patients with SLE, providing information on the optimal abstinence period to yield the *best* semen quality for subsequent fertility treatment. For patients with fluctuating semen results, concomitant semen cryopreservation should be considered to preserve the *better* quality semen before starting assisted reproductive technologies if pregnancy is planned in the future.

## 1. Introduction

Systemic lupus erythematosus (SLE) is a chronic autoimmune disorder that causes systemic inflammation, with a female-to-male incidence ratio of 9 : 1 [1]. The exact etiology of SLE remains unknown; however, common pathologies associated with SLE include inflammation, vasculitis, immune complex complement deposition, and vasculopathy [2]. The impact of SLE on male fertility has received little attention, with no study to date having used consecutive multiple semen analyses together with DNA fragmentation assays to assess sperm quality specifically for patients with SLE. This is an important issue as current therapies for SLE have been shown to reduce fertility in both males and females, particularly the use of cyclophosphamide (CY) which is associated with gonadal toxicity [3, 4]. CY suppresses spermatogenesis, which often results in abnormal semen parameters and testicular function that may or may not recover depending on the dose and duration of treatment [5]. Therefore, there is a risk for permanent azoospermia from exposure to CY. A meta-analysis of 30 studies indicated a dose-related effect of CY on the rate of gonadal failure in adult males, with a rate of 20% for a CY dose of 100–200 mg/kg (equivalent to 7.5–15 g in a 75 kg male) compared with an almost 100% failure rate with a dose >400 mg/kg (equivalent to >30 g in a 75 kg male) [6]. Of note, effects of CY exposure on gonadal failure were sustained for several years after treatment [6].

A study of 35 postpubertal males with SLE revealed abnormal semen analyses and reduced testicular volume in all patients, compared with healthy controls [7]. It was suggested that SLE disease activity causes damage to the seminiferous tubules, resulting in a significant reduction in testicular volume, made worse by treatment using intravenous CY. Another study also reported abnormal semen analyses among 4 men with SLE undergoing prednisone and/or immunosuppressive therapy who had normal erectile function, ultrasound examination of the testicles, and libido [8]. There is also evidence of an association between SLE, and its treatments, and hypothalamic-pituitary-axis dysfunction and increased gonadotrophin levels [9, 10].

Diagnosis of male infertility relies on semen analysis, based on the reference limits proposed by the World Health Organization (WHO, 5^th^ edition, 2010). According to current guidelines, semen samples for analysis should be collected after a minimum of 2 days and a maximum of 7 days of sexual abstinence. Moreover, the quality of semen cannot be reliably evaluated from a single semen sample, with 2 to 3 samples being required to obtain accurate baseline data. However, to date, there is no clear guideline regarding the effect of the period of sexual abstinence on the quality of semen and DNA fragmentation in males with SLE.

One available study investigating the effect of abstinence duration on semen quality among normozoospermic men undergoing infertility testing (*n* = 16; 11 of whom strictly complied with abstinence requirements) found that abstinence did not influence the pH, viability, morphology, or motility of the sperm [11]. However, a longer period of abstinence did improve sperm concentration and semen volume. Specifically, a 24-hour period of abstinence significantly increased the proportion of the normospermic sperm having immature chromatin, but with no effect on DNA fragmentation. No such information for males with SLE was identified.

DNA damage in sperm has been shown to reduce fertilization rates, embryo development, and pregnancy outcomes in a general population [12, 13]. Although diagnostic testing of sperm DNA fragmentation is not yet routine in most fertility clinics, there is increasing evidence that it is a valuable diagnostic tool [14]. Various optimal cutoff points for the DNA fragmentation index (DFI) have been proposed. Two studies in particular used the Halosperm G2® Sperm Chromatin Dispersion (SCD) testing kit (Halotech®, Madrid, Spain), which we used in this case report [15, 16]. Optimal DFI cutoff values of 26.1% [15] and 25.5% [16] were suggested to differentiate fertile and infertile males or for testing only males seeking fertility treatment, respectively. The impact of DFI on pregnancy outcomes was also evaluated in these studies.

Cytotoxic treatments, such as CY, are known to cause DNA damage due to their alkylating effects on the germinal epithelium [17, 18]. A recent study in mice found that CY-exposed sperm (exposure dose, 300 mg/kg for 7 days) used for *in vitro* fertilization (IVF) and intracytoplasmic sperm injection (ICSI) (22 cycles producing a total of 1212 embryos) negatively affected *in vitro* embryo development and the subsequent live birth rate, compared with the nonexposed sperm [19]. Although the mouse model is not completely translatable to humans, we are lacking direct data from human studies. For those patients who require semen cryopreservation after CY treatment, it may be necessary to utilize sperms that have potentially been damaged by CY, and, therefore, patients must be counselled appropriately regarding this risk. The WHO 2010 guideline recommends an abstinence period of 2–7 days before semen collection [11, 20] unless the epididymal function is abnormal [21]. However, there are studies that have suggested that a shorter abstinence time may improve sperm quality. One study [22], based on retrospective analysis of 9,489 semen samples, recommended an abstinence period for oligozoospermic males (non-SLE) of just 1 day, and not exceeding 10 days, due to an observed decrease in sperm motility and normal morphology. Abstinence beyond 4 days was associated with deterioration in sperm morphology. It also appears that males with reduced semen parameters may benefit from a slightly shorter abstinence period compared with normozoospermic males [22]. In fact, another study suggested that an abstinence period of 1-2 days, which is shorter than the WHO recommendation, is optimal for sperm quality and reduced DNA fragmentation [23]; however, only normozoospermic males were included in this study.

Bahadur et al. [24] reported markedly improved semen parameters (such as progressive motility, morphology, and sperm concentration) in oligozoospermic males (*n* = 73) after a short abstinence time (consecutive sample produced up to 40 minutes after the first). Effects on DNA fragmentation from the short abstinence times were not recorded, and there are few studies that have investigated the impact of a shorter period of abstinence on DNA fragmentation to benefit subfertile males. One study, however, did find that a short abstinence period (1 day) could reduce DNA fragmentation in a select group of subfertile patients. Specifically, in 35 males with increased sperm DNA fragmentation (≥30%), a 1-day abstinence protocol, with up to three ejaculation attempts, improved DNA fragmentation to normal levels in 90% of patients [25].

Current evidence regarding the optimal abstinence period is mixed, with no clear consensus to inform practice for the collection of sperm samples with higher quality in special groups of patient [26], such as males with SLE. While obtaining a semen profile with multiple semen samples may not be essential for normozoospermic males, it may be valuable for patients with SLE in whom cytotoxic treatment may lead to poor semen quality and the potential for DNA damage.

The potential impact of SLE and its associated treatment on male fertility is of major concern to young males who develop the condition, as their future fertility options may become limited. At the point of fertility assessment, a semen profile may improve the quality of sperm available for subsequent assisted reproductive technology (ART) treatment. This could be especially important as SLE treatment is likely to be ongoing, and, as such, the reduction in semen quality may persist. In this case report, we describe our approach to developing a patient-specific semen profile for a male with SLE and evaluate the effect of different periods of abstinence, over a 1-month period, on the semen quality. Based on our experience, we propose that a 4-day period of abstinence, combined multiple sample analysis, and DNA fragmentation may be clinically relevant for these patients.

## 2. Case Presentation

This male patient first received a probable diagnosis of SLE at a private hospital in 2006, at the age of 22 years, with the diagnosis confirmed 1-year later at Prince of Wales Hospital, Shatin, Hong Kong. The diagnosis of SLE was based on the presence of the following: alopecia, face rash, polyarthritis, lymphopenia, and an antinuclear antibody test titer of 1 : 640. He had a positive family history for SLE, with two paternal cousins (female) having a diagnosis of SLE. The patient had previously used ketamine, until 2010, and gave up smoking and drinking in 2014 and 2013, respectively. He had previously fathered one child, a daughter who was 8 years old, prior to starting CY treatment to manage his SLE-related symptoms.

The patient was referred to us by the IVF unit for fertility investigation in February, 2017. The patient was 33-years-old at the time of semen analysis. Data regarding the size of testes were not available as this is not a component of the routine examination in our fertility unit. Of note, the patient was fertile in spite of his early SLE diagnosis at the age of 22 years, having naturally conceived a child at the age of 25 years. He had since become infertile. In more recent years, as his SLE condition worsened, treatment with monthly intravenous (IV) CY was initiated, as follows: 6 monthly doses of 1 g in 2014 and 3 monthly doses of 1 g in 2016. Since that time, treatment with CY had been intermittent, with the last dose received in April 2016. At the time of semen analysis for this case report, the patient was being treated with corticosteroid (prednisolone) and immunosuppressant drugs, mycophenolate mofetil (MMF), and tacrolimus (TAC).

For semen analysis, the patient submitted three samples over a 1-month period, with analyses performed by an andrologist who had fulfilled the external quality assessment criteria set by the United Kingdom National External Quality Assessment Service (UK NEQAS). The samples were collected after 2, 4, and 7 days of sexual abstinence, determined as per the patient's convenience over a 1-month period. We do note that the patient had no change in his health status in the index month. Semen analysis was performed manually according to the WHO 5^th^ edition recommendations, 2010 [27], and following the advised checklist on “How to count sperm properly” published by Björndahl et al. [28]. In brief, semen collection was obtained at the clinic by masturbation, and, after liquefaction at 37°C, samples were assessed within 1 h of ejaculation. Sperm concentration and motility were assessed manually under a phase contrast microscope (Olympus BX43, Japan) at a total magnification of either ×200 or ×400. Hemocytometers, with improved Neubauer ruling, were used for the measurement of sperm concentration. To assess sperm morphology (strict Tygerber criteria), sample slides were stained with a Diff-Quik staining kit (Dade Behring AG, Switzerland) and observed under an oil immersion microscope (×100 total magnification). At least 200 sperms were counted in duplicate for each assessment.

DNA fragmentation was measured using a Halosperm G2® Sperm Chromatin Dispersion (SCD) testing kit (Halotech®, Madrid, Spain), according to the manufacturer's instructions. The detection method is based on the sperm chromatin dispersion (SCD). In brief, sperm with intact DNA will appear as a halo ring around the sperm head; different degrees of DNA fragmentation will decrease the size of the halo ring. Hydrogen peroxide- (H_2_O_2_-) induced DNA fragmentation is used as a positive control resulted in the negative halo ring, while a negative control, by omitting denaturing solution, resulted in sperm cells with the halo ring. A minimum of 300 sperms were scored per sample across two replicates, and the number of sperms with fragmented DNA (i.e., sperm without a halo or with a small or degraded halo) was represented as a percentage of the whole sample. The halo assays were performed within 1 h after the samples were submitted.

All three samples included in the analysis had normal physical properties, with the exception that the volume was below the WHO reference value of 1.5 ml. The volume did increase from 0.5 ml, 0.7 ml, and 1.0 ml with an abstinence period of 2, 4, and 7 days, respectively. The percentage of viable sperm, however, decreased as a function of increased period of abstinence from 84%, 63%, and 33% at 2, 4, and 7 days of abstinence. All samples were below the WHO reference values for motility, morphology, and total sperm count (Table 1). With a 2-day period of abstinence, the proportion of motile sperm was 28%, with the proportion decreasing to 18% and 15% for an abstinence period of 4 and 7 days, respectively. Only the sample obtained after an abstinence of 4 days had a satisfactory sperm concentration (37.3 M/ml), with the concentration after a 2- and 7-day period of abstinence being below the WHO reference value of 15 M/ml. Interestingly, the concentration gradually increased from 9.8 M/ml after a 2-day abstinence to a maximum of 37.3 M/ml after a 4-day abstinence, subsequently decreasing 18.8 M/ml after a 7-day abstinence. Normal sperm morphology also improved after a 4-day abstinence, but decreased after a 7-day abstinence, along with decreases in semen volume and concentration and total sperm count. In addition, the level of sperm DNA fragmentation was elevated after a 2-day abstinence (55.3%), reaching its lowest level after 4 days (34.7%) and then began to rise again after 7 days (38.3%). Considering our results, overall, a 4-day period of abstinence yielded the *best* semen quality, compared with an abstinence period of 2- and 7-days.

## 3. Discussion

It is generally accepted that an increase of abstinence time can improve total motile sperm count and viability and increase the level in DNA fragmentation [23, 29, 30]. However, the situation may vary in special patient groups, such as those with SLE, due to previous gonadotoxic treatment. Since DNA fragmentation assay is not routinely performed in fertility clinics, this is the first report to use multiple semen analyses, with different lengths of abstinence, together with DNA fragmentation assay to demonstrate the relationship between the period of abstinence and the level of DNA fragmentation in the semen of a patient with SLE. For this patient, a 7-day period of abstinence was associated with decreased sperm motility and slightly increased DNA fragmentation, compared with a 2- and 4-day period of abstinence. To our surprise, the shortest (2 days) abstinence time produced a sample of increased sperm motility, but with a marked increase in the level of DNA fragmentation. The current recommendation by the WHO (2010) is for semen samples to be collected after an abstinence period of between 2 and 7 days. However, in the literature, there appears to be no guideline to suggest the optimum period of abstinence in relation to semen parameters and DNA fragmentation level for patients with SLE or in those who have had prior exposure to gonadotoxic treatment. A very recent study by Tiseo et al. [31] revealed the DNA fragmentation level to be significantly higher in nonazoospermic patients with SLE when compared with a non-SLE control group. However, the period of abstinence in this study was not mentioned. In our case report, we identify that a 4-day period of abstinence, in our patient with SLE, yielded more balanced semen parameters, in terms of the level of DNA fragmentation, sperm motility, sperm concentration, and sperm morphology, with these parameters having the most influence on fertility potential. However, we do note that our case is not representative of the whole SLE population. Accordingly, we propose that a cohort study of male SLE patients would be warranted to confirm our findings. As well, semen profiles in patients with SLE would be more accurately developed from a series of semen analyses performed after different periods of abstinence and should include DNA fragmentation assay to obtain a better quality of available semen for patients with SLE undergoing subfertility treatment. Semen preservation may be applied in parallel with semen analyses in order to save the best quality sample among the multiple samples for future ART intervention.

## 4. Conclusions

A more rigorous study of the semen of patients with SLE is needed to confirm the best abstinence time. For male patients with SLE who are seeking ART treatment, it may be beneficial to develop a semen profile by obtaining multiple semen samples combining DNA fragmentation assay from the patient in order to increase the availability and quality of sperm. Cryopreservation of semen prior to SLE treatment is recommended as there is a risk of fertility impairment from current therapies.

## Figures and Tables

**Table 1:** Summary of three semen analysis reports of an SLE male from semen samples submitted after 2, 4, and 7 days of sexual abstinence.

	Sexual abstinence period			WHO (2010) lower reference limits
	2 days	4 days	7 days	
Semen parameter				
Concentration (×10^6^/ml)	9.8	37.3	18.8	15
Total sperm number in ejaculate (×10^6^)	4.9	26.1	18.8	39
Total progressive motile sperm in ejaculate (×10^6^)	1.0	2.6	1.7	—
Normal morphology (%)	1.0	2.7	2.0	4

Motility assessment				
Total motility^*∗*^ (%)	28	18	15	40
Progressive motility (%)	21	10	9	32
Nonprogressive motility (%)	7	8	6	—
Immotility (%)	72	82	85	—

Macroscopic examination				
Volume (ml)	0.5	0.7	1.0	1.5
Liquefaction (within 60 min)	Complete	Complete	Complete	—
pH	8.0	8.0	8.0	≥7.2
Colour	Normal	Normal	Normal	—
Viscosity	Slightly viscous	Slightly viscous	Slightly viscous	—
Gel clumps	None	None	None	—

Microscopic investigation				
Debris	None	None	None	—
Round cell	None	None	None	—
Red cell	None	None	None	—
Nonspecific aggregation	None	None	None	—
Agglutination of sperm	None	None	None	—

Sperm vitality				
Viable sperm (%)	84	63	33	58

DNA fragmentation				
Average DNA fragmentation (%)	55.3	34.7	38.3	—

^*∗*^Total motility = progressive + nonprogressive motility.

## Abbreviations

SLE: Systemic lupus erythematosus
CY: Cyclophosphamide
ART: Assisted reproductive technology
WHO: World Health Organization
DFI: DNA fragmentation index
SCD: Sperm chromatin dispersion
IVMP: Intravenous methylprednisolone
IVCY: Intravenous cyclophosphamide
UKNEQAS: UK National External Assessment Service.
